# Direct contact of platelets and their released products exert different effects on human dendritic cell maturation

**DOI:** 10.1186/1471-2172-9-54

**Published:** 2008-09-25

**Authors:** Hind Hamzeh-Cognasse, Fabrice Cognasse, Sabine Palle, Patricia Chavarin, Thomas Olivier, Olivier Delézay, Bruno Pozzetto, Olivier Garraud

**Affiliations:** 1 Mucosal Immunity and Pathogen Agents Group (GIMAP-EA3064), Faculty of Medicine, Jean Monnet University of Saint-Etienne, Saint-Etienne, France; 2 Auvergne-Loire Regional Blood Bank (EFS Auvergne-Loire), Saint-Etienne, France; 3 4D Multiphotonic Confocal Microscopy Platform, (Hubert Curien Laboratory and UMR CNRS 5516), Jean Monnet University of Saint-Etienne, Saint-Etienne, France

## Abstract

**Background:**

Dendritic cells (DCs) are antigen presenting cells capable of inducing innate and adaptive immune responses. According to the stimulus and their maturation state, DCs induce immunogenic or tolerogenic responses. Platelets (PLTs), which are involved in haemostasis and inflammation, can also interact with DCs. In this study, we examined the effect of PLTs on DC maturation *in vitro*. Human monocyte-derived DCs were co-cultured for 2 days with homologous PLTs either in the same well or in 0.4 μm-pore size filter-separated compartments.

**Results:**

Confocal microscopy showed the attachment of PLTs to DC membranes. The DC receptor involved in this interactions was found to be CD162. In addition, we observed that DCs co-cultured with PLTs in filter-separated compartments acquired a mature phenotype (high CD80, CD86, and intermediate CD83 expression; IL-12(p70) production; efficient stimulation of autologous CD4+ T cell proliferation), while DCs co-cultured with PLTs in the same compartment did not undergo phenotypic maturation, did not secrete IL-12(p70) or IL-1β, but instead induced moderate Th2-polarized T cell proliferation.

**Conclusion:**

These data indicate that (i) PLTs secrete a soluble DC-activating factor that was demonstrated not to be soluble CD40-Ligand (CD154; as could have been expected from *in vivo *and previous *in vitro *work) but to be nucleotide, and (ii) that cell-to-cell contact did not induce DC maturation, possibly because nucleotide release by PLTs was prevented by direct contact with DCs. This work demonstrates that PLTs are active elements of the immune system that might play a role in balancing the ability of DCs to polarize T cell responses, therefore making them critical factors in transfusion processes.

## Background

Dendritic cells (DCs) are sentinels of the immune system, involved in innate and adaptive immunity, the role of which is to guard the periphery for signs of foreign invasion. Recognition of pathogens by immature DCs is mediated by a set of receptors that includes Toll-like receptors (TLRs) [1], Fc-receptors [2], and C-type lectins [3]. Upon a "danger signal", immature DCs develop into mature immunostimulatory DCs. The DC maturation program includes a change in the expression profile of chemokine receptors, enabling the maturing DCs to migrate toward draining lymph nodes [4].

Maturing DCs overexpress costimulatory molecules (CD80, CD83, and CD86) and molecules involved in antigen presentation (Major Histocompatibility Complex class I and II) on their membranes. Matured DCs promote CD4^+ ^or CD8^+ ^T cell and B cell activation [5], and also interact with NK cells [6]. In addition, DCs can select the type of immune response by polarizing lymphocytes towards Th1, Th2, or Treg response profiles. The balance between these three types of responses depends not only on the inducing-signal, i.e., the nature of the foreign antigen [1], but also on the maturation state of DCs [7]. This aspect could be of particular importance during transfusion practices, which imply homologous cells such as platelets (PLTs).

In addition to their haemostatic role, PLTs have been shown to play a part in inflammation [8] and in innate [9] and adaptive immune responses [10]. Furthermore, DC susceptibility to PLT-derived components has already been observed [11,12], and several studies have shown that activated PLTs can modulate DC activation [10,11]. However, during transfusions, transfused homologous PLTs that are not activated and might also act on DCs.

In this work, we investigated the influence of homologous PLTs on DC activation status. We observed that PLTs co-cultured with DCs in a filter-separated compartment released nucleotides that induced maturation of DCs, as shown by an overexpression of costimulatory molecules, IL-12(p70) production, and stimulation of autologous CD4^+ ^T cell proliferation. In contrast, DCs co-cultured in direct contact with PLTs remained phenotypically immature, did not produce IL-12(p70) and IL-1β, and induced only a weak Th2-polarized T cell proliferation. These data indicate that PLTs affect DC activation differently depending on whether when they are in close contact with DCs or not.

## Methods

### Culture medium and cytokines

Both PLTs and monocyte-derived DCs were maintained in RPMI 1640 supplemented with L-glutamine (Abcys, Paris, France) and 1% penicillin-streptomycin solution (Sigma Aldrich, Saint-Quentin, France), hereafter called minimal medium. For DC differentiation, the culture medium was supplemented with 10% heat-inactivated endotoxin-free fetal calf serum (FCS; Invitrogen, Cergy Pontoise, France), recombinant human granulocyte macrophage-colony stimulating factor (GM-CSF; specific activity: 10^7 ^U/mg) and IL-4 (specific activity: 5 × 10^6 ^U/mg; Peprotech, Abcys).

### Monocyte purification and culture

Peripheral blood from healthy donors (provided by the Auvergne-Loire Regional Blood Bank) was divided into two parts, one part for monocyte purification and the other for CD4^+ ^T cell isolation. Mononuclear cells were obtained by centrifugation on Histopaque^®^1077 (Sigma Aldrich) and monocytes were depleted of NK, T, and B cells using mouse anti-CD2, CD7, CD16, CD19, CD56, and CD235a (glycophorin A) monoclonal antibodies (mAbs) and anti-mouse Ig coupled to magnetic microbeads, according to the manufacturer's instructions (Dynal/Invitrogen, Cergy Pontoise, France). The technique routinely results in > 90% purified CD14-expressing cells and cell viability was greater than 95%, as determined by Trypan blue exclusion. Monocytes (10^6 ^cells/ml) were cultured for 5 days in six-well tissue culture plates (BD Falcon™, Becton Dickinson, Le-Pont-de-Claix, France) in minimal medium supplemented with 10% FCS (Myoclone^®^, Invitrogen), recombinant human GM-CSF (400 U/ml) and IL-4 (660 U/ml), to produce DCs [13]. On days 2 and 4, cells were fed with fresh medium and cytokines. After 5 days in culture, all cells expressed CD1a, neither CD14 nor CD83, low levels of costimulatory molecules and, as described previously, CD162 [14].

### Platelet isolation

Platelet concentrates used for this study were rigorously identical as required for French transfusion purposes. Briefly, blood from volunteer healthy donors and PLT concentrate mixtures starting from buffy coats were prepared at the Auvergne-Loire Regional Blood Bank. Buffy coats were stored at 20°C without agitation for 2 h. Then, five buffy coats and a storage solution (T-sol^®^, Baxter, Maurepas, Belgium) were pooled and centrifuged. The PLT-rich supernatant was collected and leukofiltrated under pressure in order to remove residual leukocytes to below 10^5 ^leukocytes/3.5 × 10^11 ^PLTs, according to the French Blood Bank routine procedures. To minimize the transport of soluble proteins bound on PLT membranes and to avoid any alteration of PLT phenotype in our study, we used an established PLT washing method that prevents transitory platelet activation during the preparation [15]. Briefly, platelets were washed twice in Tyrode's buffer supplemented with apyrase (0.02 U/mL) and prostacyclin (0.5 mM) and resuspended in the same buffer. These procedures induce moderate PLT activation, with low CD62P expression (almost 15%), but do not interfere with transfusion grade status of purified PLTs [9].

### Dendritic cell/platelet co-culture

Co-cultures were maintained in serum-free medium in order to avoid FCS-induced PLT activation, as described by Hilf *et al*. [11]. DCs (5 × 10^5^) were co-cultured in 3 ml of minimal medium for 2 days with 1.5 × 10^7 ^homologous PLTs (DC:PLT ratio, 1:30) either in the same compartment or in 0.4-μm pore-sized filter-separated compartments in a six-well culture plate (Corning Costar/Fisher Bioblock Scientific, Illkirch, France). PLTs and DCs were not derived from the same donor in order to mimic most PLT transfusion practices in which recipients receive homologous PLTs. The 0.4-μm-pore-sized filter enables soluble factor diffusion while blocking direct cell-to-cell contact. As negative controls, DCs and PLTs were cultured alone in minimal medium without significant mortality, as assessed by Trypan blue exclusion. As positive controls, DCs were treated for 48 h with 100 ng/ml of purified soluble trimeric human CD154 (sCD154; a kind gift from Immunex-Amgen Corp., Seattle, WA, USA) or with 500 ng/ml of *E. coli *0111:B4 lipopolysaccharide (LPS; Calbiochem/VWR, Strasbourg, France) and PLTs were treated for 30 min with 1 U/ml of thrombin (Calbiochem/VWR). To determine whether soluble factors released by platelets during co-culture could be involved in DC activation, both untreated and 1% paraformaldehyde-fixed PLTs [11] were used in co-culture. In order to determine the importance of PLT-binding in DC-PLT interactions during co-culture, DCs were incubated for 1 h at 37°C prior to co-culture and during the entire co-culture period with 10 μg/ml of anti-human CD162 blocking mouse mAb [16] (PL1 clone, Immunotech Beckman-Coulter, Marseille, France). Finally, to assess the involvement of PLT-derived nucleotides (adenosine diphosphate (ADP) and adenosine triphosphate (ATP)) in DC activation during co-culture, DCs were incubated for 1 h at 37°C prior to co-culture and during the entire co-culture period with 30 μM of the broad-spectrum P2 receptor antagonist suramin (Sigma-Aldrich) [17]. Then, supernatants were recovered for cytokine/chemokine content analysis and cells were washed twice with minimal medium. Half of the DCs (i.e., 2.5 × 10^5 ^cells) was stained and analyzed either by flow cytometry or confocal microscopy. CD1a expression on DCs was confirmed after co-culture with PLTs in order to rule out any reversion of DC differentiation. The other half of the DCs was used for autologous T cell activation.

### Flow cytometry

Cells were incubated for 30 min at 4°C with 10% FCS-PBS and affinity-purified mouse mAb at the appropriate concentration or irrelevant isotype-matched mouse Igs at the same concentration and then washed. The following mAbs were used: anti-CD1a-Allophycocyanin (APCy), anti-CD40-PE, anti-CD41-FITC, anti-CD41-APCy, anti-CD62P-PE, anti-CD80-PE, anti-CD83-PE, anti-CD86-PE, anti-CD162-PE, and anti-CD209-PE (all from Becton Dickinson-PharMingen). To avoid non-specific antibody binding, all staining experiments were performed in the presence of Fc-receptor blocking reagent (Miltenyi Biotec, Paris, France). Fluorescence analysis was performed on a FACSCalibur using CellQuestPro software (Becton Dickinson-Biosciences).

### Platelet binding assay

To determine the DC receptors involved in PLT binding, DCs (2.5 × 10^5^) were incubated, or not, with 7.5 × 10^6 ^PLTs (DC:PLT ratio, 1:30) for 1 h at room temperature to allow PLT binding without affecting DC phenotype. DCs were then stained for potential PLT receptors. In addition, to investigate the involvement of CD162 and DC-SIGN in PLT binding, DCs were incubated with with 10 μg/ml of anti-human CD162 blocking mouse mAb [16] (PL1 clone, Immunotech Beckman-Coulter), 100 mM mannan (Sigma-Aldrich), or 10 μg/ml of isotype-matched irrelevant mouse antibody. Cells were then washed and incubated for 1 h at room temperature with PLTs, washed extensively to avoid non-specific binding, stained for CD1a and CD41 expression, and then analyzed by flow cytometry.

### Confocal microscopy

DCs co-cultured with PLTs in the same compartment were stained at 4°C for 30 min with mouse anti-human CD209 antibody and Alexa Fluor^® ^555 goat anti-mouse IgG (H+L) and then with anti-CD41-FITC mAb, washed and fixed at room temperature for 20 min in a 4% paraformaldehyde-PBS solution. Stained cells were washed and resuspended in Vectashield^® ^mounting medium (Vector Laboratories Ltd., Abcys), mounted on a glass slide, and covered by a cover slip. Visualization was performed on a TCS SP2 Leica confocal microscope equipped with an AOBS and a 63 ×/1.4 oil-immersion objective lens with a 2.2 zoom (all from Leica Microsystèmes SAS, Rueil-Malmaison, France). Images were acquired using a dual-band 488-nm argon and 543-nm helium/neon laser and were collected using Leica Confocal Software.

### Cytokine analysis

Tumor necrosis factor α(TNF-α), IL-10, IL-12(p70), IL-1β, and RANTES content in the supernatants from co-cultured cells or cells cultured alone, and in the presence or absence of sCD154 or LPS, was measured using Luminex^® ^technology (Beadlyte^® ^Multiplex Testing Service, Upstate, Dundee, UK). Soluble CD62P, sCD40L, IL-2, and IL-4 content in culture supernatants was measured by specific ELISA using commercial kits (R&D Systems Europe Ltd, Lille, France) according to the manufacturer's instructions.

### RANTES mRNA analysis

DCs and PLTs were cultured separately or together for 2 days as described in "dendritic cell/platelet coculture" section. Cells were collected and mRNA was extracted using a Genelute direct mRNA miniprep kit (Sigma-Aldrich) according to manufacturer's instructions. After DNAse I treatment, 1 mg mRNA was retro-transcribed with M-MuLV reverse transcriptase (Promega, Charbonières, France) and fragments were amplified with Taq polymerase (Sigma-Aldrich) using primers specific for human RANTES and for β-actin (R&D Systems Europe Ltd, Lille, France), as described previously [18]. Expected product lengths were 320 bp for RANTES internal positive control, 198 bp for RANTES complementary DNA (cDNA), and 634 bp for β-actin cDNA. Amplification products were resolved by electrophoresis and product sizes were verified against a 100 bp DNA ladder (Invitrogen).

### Autologous T cell activation

For each experiment, CD4^+ ^T cells were purified from the mononuclear cells of the same blood sample from which the monocytes were derived using the Dynabeads^® ^MyPure™ CD4 T Cell Kit 2 (For Untouched Human Cells; Dynal/Invitrogen). Briefly, CD4^+ ^T cells were depleted of NKs, monocytes, and B cells using mouse anti-CD8, CD14, CD16 (specific for CD16a and CD16b), CD19, CD36, CD56, CDw123, and CD235a (glycophorin A) mAbs and anti-mouse Ig coupled to magnetic microbeads, according to the manufacturer's instructions. The technique routinely results in >95% purified CD4-expressing cells and cell viability was greater than 95%, as determined by Trypan blue exclusion. After washing, cells were frozen until use. Once thawed, CD4^+ ^autologous T cells were labeled with carboxyfluorescein diacetate succinimidyl ester (CFSE, Invitrogen). Cells were first washed in RPMI1640 without FCS and then incubated with 5 μM of CFSE at 37°C for 20 min. The reaction was stopped by adding FCS. Cells were washed extensively and resuspended at 2.5 × 10^6 ^cells/ml in 10% FCS-supplemented RPMI1640. In parallel, DCs from the different co-culture conditions with PLTs were resuspended at 2.5 × 10^5 ^cells/ml and co-cultured with 2.5 × 10^6 ^autologous CD4^+ ^T cells (DC:T cell ratio = 1:10) for 7 days in individual wells of 12-well round-bottom plates. Cells were then harvested and T cell proliferation was analyzed immediately by flow cytometry measuring the CFSE fluorescence intensity. The percentage of CFSE^low ^T cells in co-culture with DCs was normalized to percentage of CFSE^low ^in untreated T cells (control) according to the following formula: ((% CFSE^low^_co-culture _- % CFSE^low^_control_)/% CFSE^low^_control_) × 100. Untreated CD4^+ ^T cells were used as negative controls and T cells stimulated with 2.5 μg/ml phytohemagglutinin (PHA) and 100 U/ml human recombinant IL-2 for 48 h were used as positive controls. Finally, supernatants were collected in order to measure secreted IL-2 and IL-4, cytokines typical of Th1 and Th2 responses, respectively.

### Statistical analysis

Inter-experiment comparisons were performed by Mann-Whitney U test (Statistica-StatSoft, Maisons-Alfort, France).

## Results

### Platelet binding to dendritic cells

**Figure 1:**
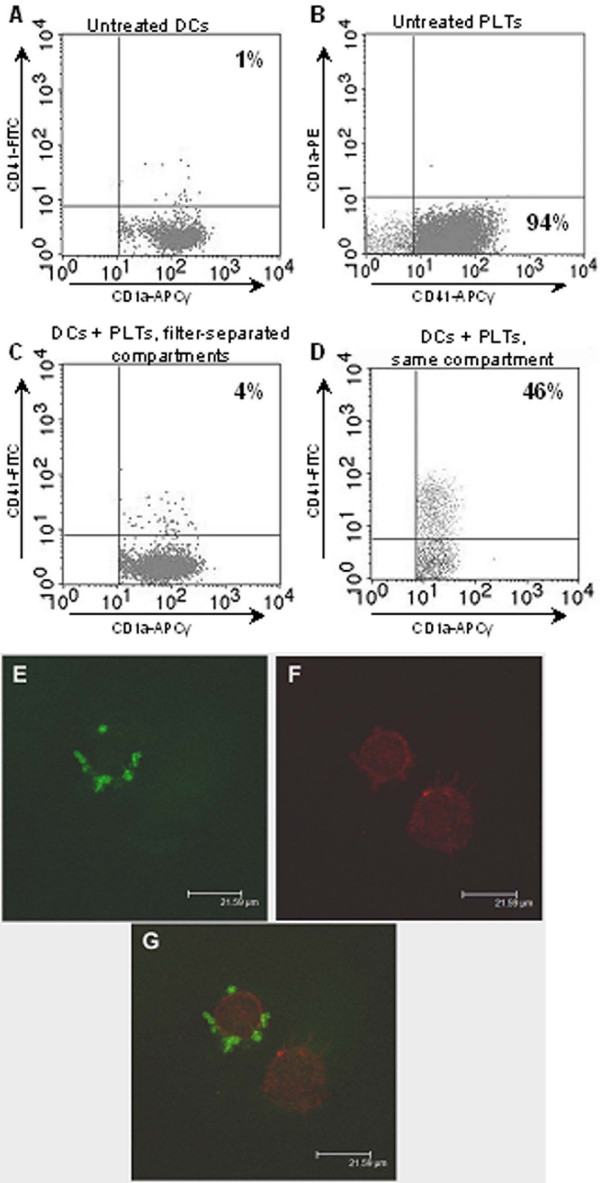
**Platelet binding to dendritic cells**. Platelet (PLT) binding to dendritic cells (DCs) was evaluated by flow cytometry (A-D) or by confocal microscopy (E-G). DCs and PLTs were cultured for 48 h alone (A-B) or together, either in two 0.4-μm pore-sized filter-separated compartments (C) or in the same compartment (D). DCs and PLTs were stained for CD1a and CD41, respectively, and analyzed by flow cytometry (A-D). For PLTs cultured alone, flow cytometry plots are not gated on any marker. For DCs, flow cytometry plots are gated on CD1a^+ ^events only. Plots are representative of five independent experiments. (E-G) DCs cultured with PLTs in the same compartment were stained for CD209 and CD41 and analyzed by confocal microscopy (E, anti-CD41-FITC; F, anti-CD209- Alexa Fluor^® ^555 and G, anti-CD41-FITC, and anti-CD209- Alexa Fluor^® ^555). The confocal microscopy photograph is representative of three independent experiments.

As expected, PLTs did not express CD1a (Figure 1B). The PLT-specific marker CD41 was expressed neither by untreated DCs (Figure 1A) nor by DCs co-cultured with homologous PLTs in filter-separated compartments (Figure 1C), confirming that no soluble CD41 is released from PLTs and subsequently binds to DCs. However, nearly half of the DCs co-cultured with PLTs in the same well expressed CD41 (p < 0.05), suggesting that PLTs were binding to the DC membrane (Figure 1D). To confirm this observation, we analyzed the expression of CD41 and CD209 (DC-Specific ICAM-3 Grabbing Nonintegrin, DC-SIGN) on PLTs and DCs co-cultured in the same well by confocal microscopy (Figure 1E–G). An attachment between PLTs and DCs was clearly visible (Figure 1G). The PLTs were tightly bound to the DCs and extensive washing of the cell preparation prior to confocal microscopy and cytometry analysis did not separate the two cell types.

**Figure 2:**
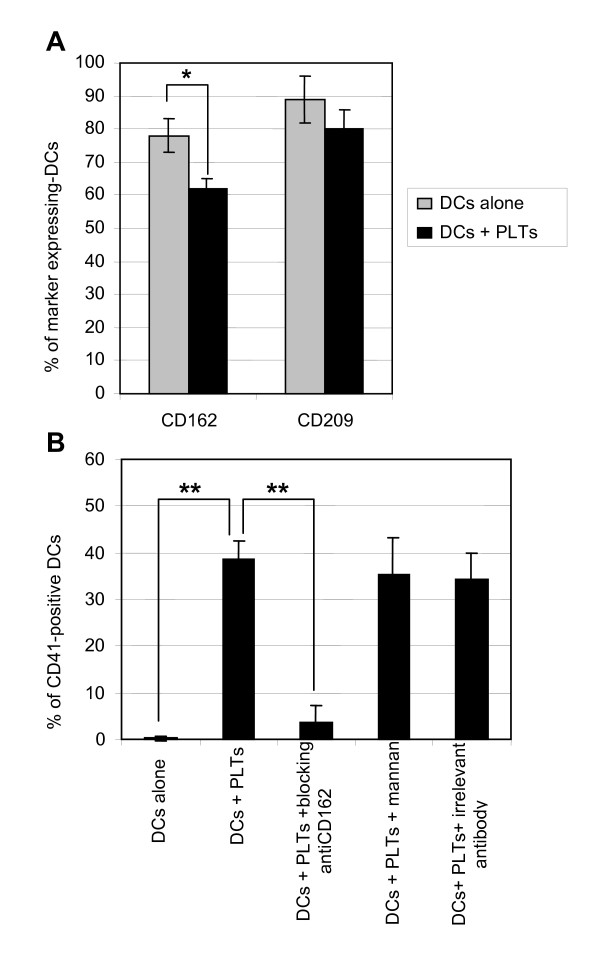
**Potential dendritic cell receptors for platelets**. Dendritic cells (DCs) were incubated with or without platelets (PLTs) for 1 h (A), or pre-incubated for 1 h with an anti-CD162 blocking antibody, mannan, or an irrelevant antibody and then incubated for 1 h with PLTs (B). DCs were then stained for CD162 and CD209 (A) or for CD41 (B) and analyzed by flow cytometry after gating on CD1a^+ ^cells only. The results presented are mean values ± SD of five independent experiments; percentage of marker expression in each experiment was assessed in triplicate. Asterisk indicates statistical significance a p < 0.05 (*) or < 0.005 (**) by Mann-Whitney U test.

Therefore, we investigated which PLT and DC receptors were involved in PLT-DC binding. DCs were incubated with or without PLTs for 1 h to allow PLT binding without affecting DC phenotype. DC were washed extensively and stained for several potential PLT receptors. We could evidence that in the presence of PLTs, the percentage of CD162-expressing DCs decreased significantly (p < 0.05) from 78 ± 5% to 62 ± 3%, whereas the percentage of CD209-positive DCs did not change significantly although a small diminution seemed to occur (Figure 2A). This observation suggests that CD162 molecules on DCs are occupied by PLTs.

In order to confirm the involvement of CD162 in PLT-DC binding, we incubated DCs with blocking anti-CD162 mAb prior to incubation with PLTs. We observed that the percentage of DCs expressing CD41, reflecting the percentage of DCs with PLTs bound to their membrane, decreased significantly (4 ± 3%) as compared to DCs incubated with PLTs without anti-CD162 preincubation (39 ± 4%; p < 0.005; Figure 2B). In contrast, preincubation of DCs with either 100 mM of mannan or 10 μg/ml of an irrelevant antibody did not alter the percentage of CD41-positive DCs (38 ± 8%). This confirms that PLTs bind to DCs, at least in part, *via *the CD162 receptor on DC and that CD209 does not participate significantly to PLT-DC binding.

### Dendritic cell activation by platelets

**Figure 3:**
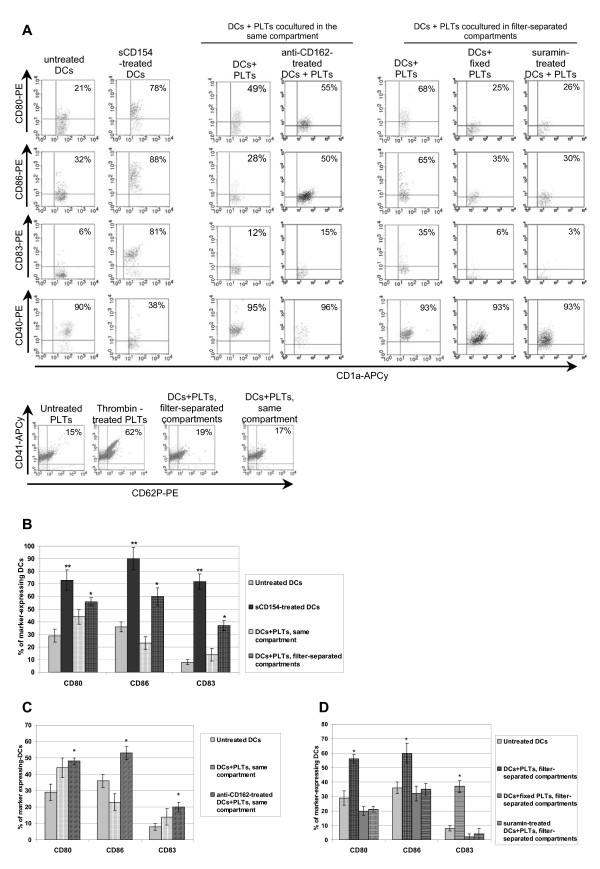
**Activation status of dendritic cells and platelets during co-culture**. Dendritic cells (DCs) were cultured for 48 h alone (untreated), with sCD154, or with platelets (PLTs) either in two 0.4-μm pore-sized filter-separated compartments or in the same compartment. To evaluate the contribution of soluble factors released by PLTs on DC activation, PLTs were used either untreated or following fixation with paraformaldehyde. DCs used in co-cultures were untreated, preincubated with blocking anti-CD162 mAb to prevent PLT binding when placed in the same well, or preincubated with suramin in order to block ATP receptors. DCs were then stained for CD1a and CD80, CD86, CD83, or CD40 and analyzed by flow cytometry. The flow cytometry plots are gated on CD1a^+ ^cells only. PLTs cultured without DCs (untreated), after thrombin activation, and after co-culture with DCs in separate or the same compartments were stained for CD41 and CD62P. (A) Representative results of five independent experiments and (B-D) mean values ± SD of three independent experiments for suramin, blocking anti-CD162 mAb, and fixed PLTs conditions, and of five independent experiments for the other conditions. Mean values for activation marker expression by DCs co-cultured with PLTs are summarized (B) and developed for co-culture in the same well (C) and for filter-separated co-culture (D). Percentages of marker expression in each experiment were assessed in triplicate. Asterisks indicates statistical significance between assay and untreated DCs a p < 0.05 (*) or < 0.005 (**) by Mann-Whitney U test.

After 5 days in culture, monocytes differentiated into viable typical DCs with a classical non-activated phenotype, as shown in Figure 3A, displaying cytometry plots of a representative experiment. Culture of DCs in minimal medium for 2 days did not induce significant modification of CD1a or costimulatory molecule expression. As well, minimal medium did not induce a higher level of PLT activation as compared to freshly isolated PLTs, the latter displaying only a weak CD62P expression related to the isolation process (Figure 3A). As a positive control for DC activation, we treated DCs with soluble CD154 (sCD154) and observed a significant increase in the mean expression of CD80, CD86, and CD83, which reached 73 ± 8%, 90 ± 9%, and 72 ± 6%, respectively (p < 0.05; Figure 3B). When PLTs were co-cultured with DCs in filter-separated compartments, we observed a notable proportion of CD80-, CD86-, and CD83-positive DCs (56 ± 3%, 60 ± 7%, and 37 ± 4%, respectively), which differed significantly from the untreated control (p < 0.05), demonstrating that PLTs induce moderate but significant DC activation (Figure 3B). However, when PLTs were co-cultured in direct contact with DCs, we observed only a slight increase in the proportion of activated DCs, which did not differ significantly from the untreated controls in mean expression of CD80 (44 ± 6%), CD86 (21 ± 5%), and CD83 (14 ± 5%; Figure 3B). Therefore, we can assume that PLTs release soluble factors that induce DC activation and that cell-to-cell contact between PLTs and DCs does not promote DC activation. Indeed, when DCs were incubated with a blocking anti-CD162 mAb prior to and during co-culture with PLTs, there was a significant increase in the mean expression of CD80 (48 ± 2%), CD86 (53 ± 4%), and CD83 (20 ± 3%) on DCs (Figure 3C), demonstrating that PLT binding to DCs prevents the former from activating the latter.

Moreover, PLTs fixed in 1% paraformaldehyde did not induce a significant increase in CD80, CD86, and CD83 expression (respectively, 20 ± 3%, 32 ± 5%, 2 ± 2%), confirming the involvement of a soluble factor in DC activation during filter-separated co-culture with PLTs (Figure 3D). Given that PLTs release large amounts of sCD154 during their storage [19] or upon activation [20], we investigated whether the DC activation that we observed could have been induced by PLT-derived sCD154. However, we did not detect any sCD154 in the supernatants from PLTs and DCs cultured separately or from co-cultures of these two cell types (ELISA data not shown). We did not observe a decrease in CD40 expression by DCs either, indicating that the absence of sCD154 in the supernatant could not be explained by its binding to the DC membrane via CD40 (Figure 3A). However, the lack of detectable sCD154 could have been related to the non-activated status of the PLTs. Indeed, in our study, PLTs were not submitted to any activation stimulus and did not exhibit an activated phenotype after co-culture with DCs (Figure 3A).

Nucleotides such as ADP and ATP, which is known to induce DC activation, are present in large amounts in PLTs [17,21]. In order to investigate whether PLT-derived nucleotides might be involved in DC activation in our system, DCs were incubated with suramin, a broad-spectrum P2 receptor antagonist, prior to and during filter-separated co-culture with PLTs. We found that suramin-treated DCs displayed an activation phenotype comparable to that of untreated DCs (i.e., low mean expression of CD80 (21 ± 2%), CD86 (35 ± 4%), and CD83 (4± 4%); Figure 3D). Thus, during filter-separated co-culture, PLTs release nucleotides that can activate DCs. Interestingly, DCs that were in close contact with PLTs expressed more CD80 than CD86 (44 ± 6% *vs*. 21 ± 5%, respectively), whereas DCs activated by PLTs through a filter expressed more CD86 than CD80 (60 ± 7% *vs*. 56 ± 3%, respectively; Figure 3B).

Thus, filter-separated co-culture of PLTs with DCs does not activate PLTs but induces PLT release of nucleotides capable of maturing DCs. In contrast, when these two cell types are co-cultured in the same well, PLTs are maintained in a steady state, they do not release nucleotides and DC activation is not induced.

### Cytokine production

**Figure 4:**
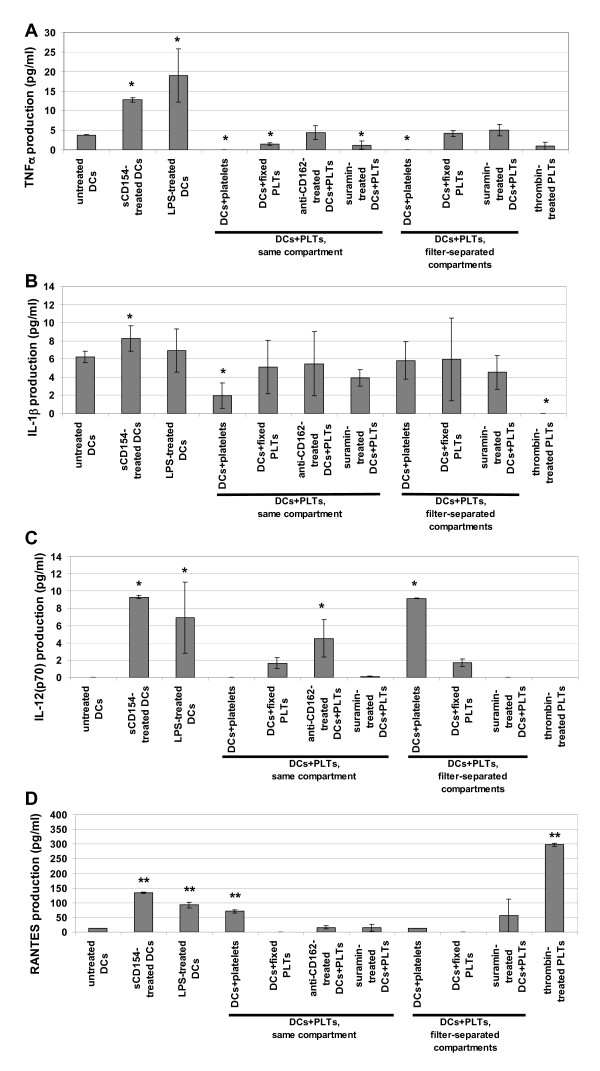
**Cytokine and chemokine production in dendritic cell-platelet co-culture**. Dendritic cells (DCs) were cultured for 48 h alone (untreated), with sCD154, or with untreated or fixed platelets (PLTs) either in two 0.4-μm pore-sized filter-separated compartments or in the same compartment, with or without blocking anti-CD162 mAb or suramin. Thrombin-treated PLTs were used as positive controls for cytokine and chemokine release by activated PLTs. Supernatants were collected and TNFα (A), IL-1β (B), IL12-p70 (C), and RANTES (D) production was assessed by the Luminex^® ^technology. Histograms represent mean values ± SD of three independent experiments, with each experiment assessed in triplicate. Asterisks indicates statistical significance between assay and untreated DCs a p < 0.05 (*) or < 0.005 (**) by Mann-Whitney U test.

We next investigated whether PLTs could modulate DC function. Using the Luminex^® ^technology, we assessed the release of the main pro-inflammatory cytokines TNF-α, IL-1β, and IL12-p70, in addition to RANTES which can also be released by PLTs, by DCs cultured alone (negative control), by sCD154 or LPS-treated DCs (positive controls), and by DCs co-cultured with PLTs (Figure 4). We found that filter-separated co-culture of PLTs with DCs, i) inhibited TNF-α production; this inhibition was dependent on the release of nucleotides by PLTs because paraformaldehyde fixation of PLTs and suramin treatment of DCs restored the initial IL-1β production (Figure 4A); ii) did not modify IL-1β (Figure 4B) or RANTES production significantly (Figure 4D); but, iii) did induce significant IL-12(p70) production dependent on the release of nucleotide by PLTs (PLT fixation and suramin treatment reduced IL-12(p70) production to the level of the negative control; Figure 4C). Moreover, IL-10, sCD154, and soluble CD62P (sCD62P) were not detected in co-culture supernatants (data not shown). Therefore, we found that the activated phenotype of DCs induced by filter-separated co-culture with PLTs was associated with the functional activation of DCs, leading to IL-12(p70) production.

On the other hand, PLTs co-cultured in direct contact with DCs did not trigger IL-12(p70) production, unless this contact was prevented by a blocking anti-CD162 mAb (Figure 4C) and inhibited TNF-α (Figure 4A) and IL-1β production (Figure 4B); however, their mechanisms of inhibition of appear to be different. PLT fixation and suramin-treatment of DCs did not restore TNFα production, whereas the blocking anti-CD162 mAb did, showing that PLT binding to DCs, and not factor release by PLTs, is crucial for inhibiting the production of TNFα (Figure 4A). As for the inhibition of IL-1β production, PLT fixation, treating DCs with suramin and blocking anti-CD162 DC treatment returned IL-1β production to its original level, suggesting that upon binding to DCs, PLTs release nucleotides that inhibit IL-1β production (Figure 4B).

**Figure 5:**
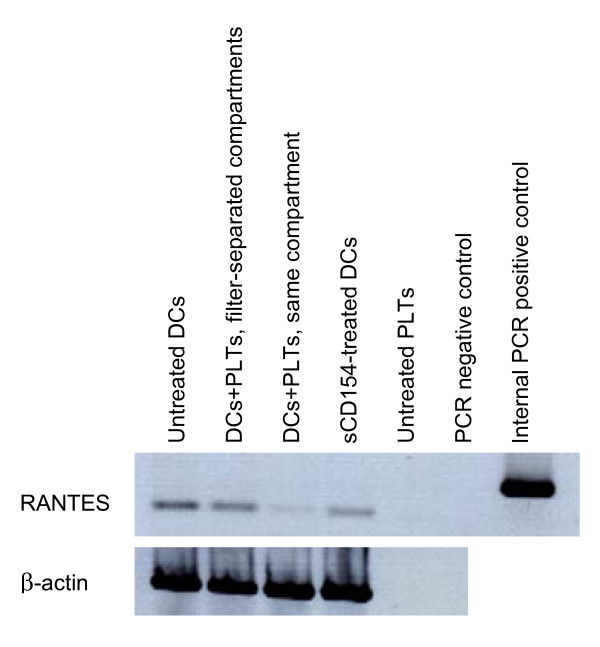
**RANTES mRNA analysis in dendritic cell-platelet co-culture**. Dendritic cells (DCs) were cultured for 48 h with sCD154 or with platelets (PLTs) either in two 0.4-μm pore-sized filter-separated compartments or in the same compartment. As culture negative controls, DCs or PLTs were cultured alone for 48 h (untreated DCs, untreated PLTs). The positive PCR control consisted of a synthetic double-stranded DNA provided by the manufacturer, the negative PCR control consisted of water instead of cDNA template for PCR. Data are representative of three independent experiments.

These observations indicate that intimate contact between PLTs and DCs does not promote DC polarization towards a proinflammatory response, even if we did observe a significant increase in RANTES production (Figure 4D). However, under these conditions, the level of RANTES production was far less than the level induced by sCD154-treatment of DCs or thrombin-treatment of PLTs, and was prevented by paraformaldehyde fixation of PLTs. These data suggest that RANTES is released by PLTs (Figure 4D; in agreement with the results of Danese *et al*. [22]). We investigated whether the increase in RANTES in the co-culture supernatant could be due to production by DCs. We extracted RANTES mRNA from DCs and PLTs cultured alone and from DCs and PLTs co-cultured together or in filter-separated compartments. There was no increase in RANTES mRNA in DCs cultured with PLTs in the same compartment as compared to the other culture conditions (Figure 5). Interestingly, DCs in direct contact with PLTs synthesized even less RANTES mRNA, suggesting that RANTES found in the supernatant from direct contact co-culture of DCs and PLTs is released by PLTs rather than by DCs and that contact between these two cell types may reduce RANTES mRNA synthesis in DCs.

### Autologous T cell activation

**Figure 6:**
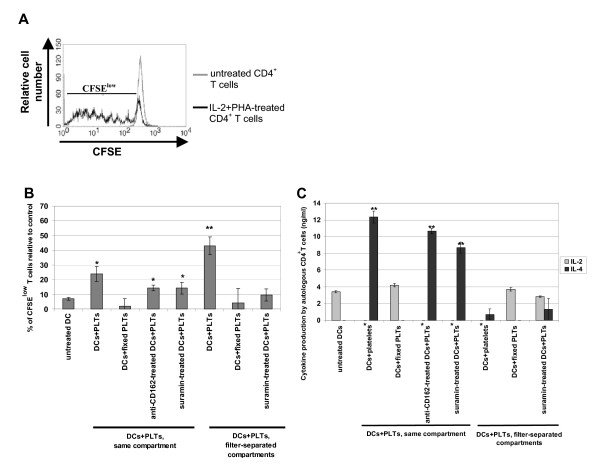
**Autologous T cell activation by dendritic cells co-cultured with platelets**. Untreated dendritic cells (DCs) or DCs co-cultured for 48 h with platelets (PLTs) either in the same well or in filter-separated compartments were co-cultured with autologous CD4^+ ^T cells stained with carboxyfluorescein diacetate succinimidyl ester (CFSE). After 7 days of co-culture, T cell proliferation was determined by the percentage of CFSE^low ^cells (B) and culture supernatants were recovered and analyzed for IL-2 and IL-4 content (C). (A) Represents typical FACS histograms of CFSE staining of untreated or IL-2^+ ^PHA-stimulated CD4^+ ^T cells. (B) Percentage of CFSE^low ^T cells in co-culture with DCs was calculated according to the following formula described in the Methods section. (B, C) values are mean ± SD of three independent experiments, with each experiment assessed in duplicate. Asterisks indicates statistical significance between assay and untreated DCs at p < 0.05 (*) or < 0.005 (**) by Mann-Whitney U test.

Next, we investigated the capacity of DCs co-cultured with PLTs to induce autologous CD4^+ ^T cell proliferation in a mixed leukocyte reaction using CFSE labeling. CFSE is a stable cytoplasmic fluorescent dye that segregates equally between daughter cells upon cell division. Thus, the relative intensity of the dye decreases and dividing cells are characterized by their low CFSE content (CFSE^low ^cells). As a positive control, we stimulated CD4^+ ^T cells with IL-2 and PHA and observed an increase in CFSE^low ^cells as compared to untreated T cells (Figure 6A). We assessed the DC induction of T cell proliferation as described in Methods section.

We observed that PLTs co-cultured in direct contact with DCs, although they induced neither significant overexpression of costimulatory molecules nor IL-12(p70) production by DCs, could stimulate DCs enough to trigger a significant T cell proliferation as compared to DCs alone, 24 ± 5% and 7 ± 1% respectively (Figure 6B). This T cell proliferation was prevented when PLTs were fixed with paraformaldehyde but not when DCs were treated with suramin or blocking anti-CD162 mAb (Figure 6B), indicating that (i) DC stimulation by PLTs in the same well occurs *via *the release of a soluble factor, (ii) nucleotides such as ADP or ATP are not involved, and (iii) PLT binding on DCs is not critical for the release of this factor. In addition, during filter-separated co-culture, PLTs stimulated DCs more efficiently in terms of induction of T cell proliferation (43 ± 10%; Figure 6B), in agreement with the phenotypic activation of DCs and IL-12 production. T cell proliferation induced by DCs from filter-separated co-culture with PLTs relied on PLT-release of nucleotides, because PLT fixation and suramin-treatment of DCs prevented T cell proliferation.

Finally, we checked IL-2 and IL-4 production during autologous T cell activation in order to evaluate Th-polarization. Under each condition that induced efficient T cell proliferation, IL-2 production was completely abolished and a significant amount of IL-4 was produced (Figure 6C), suggesting Th2 polarization of T cells by PLT-stimulated DCs. However, while DCs from filter-separated co-culture with PLTs could induce T cell proliferation, significant IL-4 production paralleling the inhibition of IL-2 production was not detected, implying the involvement of other cytokines. Nevertheless, we can hypothesize that the inhibition of IL-2 production would favor a Th2 rather than a Th1 response.

Taken together, these observations suggest that direct contact between PLTs and DCs does not induce a phenotypic activation of the DCs but does polarize DCs to become Th2-inducing cells, whereas filter-separated PLTs induce a functional maturation of DCs marked by IL-12 production and inhibits DC polarization toward Th1-inducing cells.

## Discussion

Platelets express numerous cell surface molecules, such as intercellular adhesion molecule (ICAM)-2, CD62P, and a large range of glycoproteins that play a part in cell-to-cell interactions, including binding. Indeed, previous studies have shown that PLTs bind to T lymphocytes *via *a CD11a/ICAM-2 interaction [23], and bind to neutrophils and monocytes *via *CD62P/CD162 interaction [24,25].

We observed efficient binding of PLTs to DCs when these two cell types were cultured together, with nearly half of the DCs bearing PLTs (Figure 1D). We also showed that DCs interact with PLTs *via *CD162 but not *via *CD209 (Figure 2B). Although CD209 has been shown to interact with ICAM-2 during DC emigration from blood [26], we did not detect any significant binding of PLT ICAM-2 to CD209-expressing DCs. This could be explained by the sialylation of ICAM-2 on platelets, which has been reported to decrease the efficiency with which PLTs bind to CD209 [27]. In addition, PLTs display numerous membrane glycoproteins [28] and DCs display many membrane lectins [3] that could also be involved in PLT-DC binding.

Further, we investigated the nature of DC-PLT interactions using two co-culture models: filter-separated and direct-contact co-culture. Filter-separated co-culture of these two cell types resulted in DC activation accompanied by significant overexpression of costimulatory molecules that could have been influenced by the release of a soluble factor by PLTs (Figure 3D). Interestingly, Kissel *et al*. [29] reported that PLTs, activated or not, could not induce DC maturation. It is possible that their observations were the result of a lack of reversion in DC activation status rather than the inhibition of DC activation, as their DCs were activated with LPS prior to co-culture with PLTs.

*In vivo *studies have shown that PLTs can recruit leukocytes in murine models of glomeruli inflammation [30], allergic lung inflammation [31], contact hypersensitivity [32], and acid-induced acute lung injury [33], and that this recruitment was due to CD62P expressed by activated PLTs. Similarly, Elzey *et al*. demonstrated *in vivo *that the expression of CD154 on PLTs was required to induce B cell isotype switching, augment CD8^+ ^T cell responses, and induce DC maturation in mice [10]. Langer *et al*. also demonstrated PLT-induced DC activation *in vitro*, but the PLT activation state was not discussed in this report [34]. These observations underline the fact that activated PLTs can generate immune responses.

Because PLTs have been described to liberate large amounts of sCD154, representing most of the circulating sCD154 [35,36], we investigated whether DC activation could be related to PLT-derived sCD154, as has been shown in other studies of DCs [37], B cells [38], and T cells [10]. We also searched for PLT-derived sCD62P in co-culture supernatants, because sCD62P has been shown to induce monocyte differentiation into dendritic-like cells [39] and to induce polymorphonuclear cell activation [40]. In our culture conditions, no sCD154 or sCD62P was detected (data not shown), suggesting the involvement of other PLT-derived factor(s). In fact, previous studies showing that PLT-derived sCD154 induced leukocyte activation used thrombin-activated PLTs. In our hands, no PLT activation was observed during filter-separated co-culture, which could explain the lack of sCD154 release. These observations agree with those of Hagihara *et al*., who showed that an unidentified soluble factor (which was not sCD154), excreted by high shear-exposed platelets could mature DCs *in vitro *[41]. Indeed, PLTs contain many soluble factors that could play a role in DC maturation, such as growth factors, Platelet Factor 4, β-thromboglobulin [42], ATP [17], and proinflammatory chemokines [43]. When we treated DCs in filter-separated co-culture with PLTs with suramin, a broad-spectrum P2 receptor antagonist, costimulatory molecule expression on DCs was restored to its initial level, supporting the hypothesis that PLT-derived nucleotides are involved in DC activation (Figure 3D).

Quantifying ATP release by PLTs would be particularly useful for our model. However, measuring ATP released into culture supernatants is not straightforward – P2 receptors can bind up nucleotides in the medium and ecto-nucleotidases on DCs can rapidly hydrolyze extracellular ATP [44]. Furthermore, ATP measurement requires specific bioluminescence assay equipment [45]. However, low concentrations of extracellular ATP were shown to increase CD80, CD86, and CD83 expression on DCs, which is consistent with our results [21].

The activated phenotype exhibited by DCs in filter-separated co-culture with PLTs was associated with their functional activation, as assessed by their increased IL-12(p70) production (Figure 4C) and their induction of autologous T cell proliferation. The T cell proliferation was prevented by suramin-treatment, confirming the role of PLT-derived nucleotides in DC activation (Figure 6B).

Our results contrast with those of Kissel *et al*., who reported PLT-induced inhibition of IL-12(p70) production. However, Kissel *et al*. used LPS to stimulate IL-12(p70) production by DCs. Nonetheless, we must consider that PLTs express functional TLR4, which could explain the differential modulation of DC maturation [9,46]. In contrast to the observed increase in IL-12(p70) production, we observed no significant increase in TNFα or IL-1β production related to PLT-induced DC activation, as was expected [47,48]. These observations correlate partially with those of La Sala *et al*., who demonstrated that low doses of extracellular ATP induced a phenotypic activation of DCs but inhibited LPS- and sCD154-dependent production of IL1-β, TNF-α, and IL-12(p70) [21]. These discrepancies concerning IL-12(p70) could be due to variations in the dosage of ATP: *La Sala et al*. used low concentrations of extracellular ATP while we relied on PLT release of ATP, which is known to be substantial. Moreover, we cannot exclude the possibility that PLTs released other soluble factors(s), such as arachidonic acid metabolites [49], that could modulate the effects of ATP on DC activation, as has been observed for prostaglandin D2 in the inhibition of Langerhans cell migration [50].

In addition, we observed that co-culture of PLTs with DCs, either in direct or indirect contact, did not induce PLT activation and no sCD154 was released into the supernatants. Yet, we postulate that PLTs cultured in a separate compartment from DCs, while not displaying an activated phenotype, release nucleotides that activate DCs. Indeed, we showed that upon a short-delay storage (1–2 days), PLTs released several factors such as PDGF-AA and TGF-β [19] without any significant change in CD62P expression. Thus, we can assume that non-activated PLTs release baseline amounts of nucleotide, such as ADP and ATP, which can be prevented by direct contact with DCs.

Direct contact between these two cell types seems to prevent DC maturation, as no significant increase in costimulatory molecule expression by DCs during co-culture (Figure 3C). Notably, under these conditions DCs express higher levels of CD80 than CD86, which seems to be related to a "tolerogenic" DC phenotype. It was previously shown that CD80 and CD86 differentially modulate the suppressive function of regulatory T cells, with CD80 enhancing the Treg suppressive function and CD86 inhibiting the Treg suppressive function [51]. Without going so far, these observations suggest that direct contact between PLTs and DCs does not result in DC activation, but may promote a DC phenotype related more to a "tolerogenic-like" state than to an "activating" state.

The non-activated phenotype observed when DCs were in direct contact with PLTs was associated with a lack of IL-12(p70) production and the inhibition of IL-1β and TNFα production (Figures 4 A-B). However, mechanisms for inhibiting IL-1β and TNFα production seemed to differ from one another. Whereas the inhibition of TNFα production relied on PLT binding to DCs exclusively (Figure 4A), inhibition of IL-1β production seemed to be based on nucleotide release by PLTs upon binding to DCs (Figure 4B).

We noted a significant increase in the amount of RANTES in the direct co-culture supernatant, but less so than that observed when DCs were treated with sCD154 or when PLTs were treated with thrombin (Figure 4D). This increase in RANTES was linked to a slight decrease in RANTES mRNA in DCs (Figure 5). These observations, associated with the lack of RANTES when PLTs were fixed with paraformaldehyde, strongly suggest that RANTES was released by platelets upon contact with DCs. Finally, we observed that while DCs in direct contact with PLTs neither exhibited an activated phenotype nor produced IL-12(p70), they could stimulate autologous T cell proliferation, although to a lesser extent than DCs cultured separately from PLTs (Figure 6B). However, we found that IL-2 production was inhibited and IL-4 secretion was increased when DCs were in close contact with PLTs prior to the mixed lymphocyte reaction (Figure 6C), suggesting that these DCs were polarized to induce Th2 rather than Th1 responses. As for DCs in filter-separated co-culture with PLTs, although no significant IL-4 production was observed, the inhibition of IL-2 production favors Th2 polarization. These observations agree in part with those of La Sala *et al*. who demonstrated that ATP-activated DCs produced lower amounts of IFN-γ and higher levels of IL-4 [21]. However, the mechanism involved in DC-induced T cell proliferation seems to be different depending on DC were whether in direct contact or filter-separated from PLTs. Although a soluble factor was involved (PLT fixation inhibited IL-4 and restored IL-2 production), it does not appear that this factor was ATP because suramin-treatment of DCs did not inhibit IL-4 production. Moreover, treatment with blocking anti-CD162 mAbs did not induce the same cytokine profile as culturing the two cell types in separate compartments, suggesting that the DC micro-environment might influence factor release by PLTs and indicating an increasingly complex pattern of interaction between the two cell types.

## Conclusion

In conclusion, we demonstrated that PLTs bind to DCs *via *the CD162 receptor and that cell-to-cell contact between PLTs and DCs and filter-separated co-culture have opposing effects on DC maturation. During culture in separated compartments, non-activated PLTs spontaneously release nucleotides (but not sCD154) that induce DC functional activation. Conversely, by preventing the release of nucleotides by PLTs, direct contact between DCs and PLTs neither activates PLTs nor promotes DC activation but induces polarization towards a Th2 response. This work emphasizes the importance of continued study of the interactions between PLTs and DCs, key elements to be taken into account in transfusion practices.

## Abbreviations

APCy: allophycocyanin; ADP: adenosine diphosphate; ATP: adenosine triphosphate; CFSE: carboxyfluorescein diacetate succinimidyl ester; DC: dendritic cell; FCS: fetal calf serum; ICAM: intercellular adhesion molecule; IL: interleukin; imDC: immature DC; mAb: monoclonal antibody; MHC: major histocompatibility complex; PHA: phytohemagglutinin; PLT: platelet; sCD62P: soluble CD62P; sCD154: soluble trimeric human CD154; TLR: toll-like receptor.

## Authors' contributions

HHC conceived of the study, performed the research, and wrote the manuscript. FC participated in the platelet and dendritic cell co-culture and in cytokine quantification by ELISA. SP and TO participated in the confocal microscopy analysis. PC participated in platelet preparation. OD participated in the flow cytometry analysis. BP and OG participated in the coordination of the study and helped to draft the manuscript. All authors read and approved the final manuscript.
